# Cationized Magnetoferritin Enables Rapid Labeling and Concentration of Gram-Positive and Gram-Negative Bacteria in Magnetic Cell Separation Columns

**DOI:** 10.1128/AEM.00720-16

**Published:** 2016-05-31

**Authors:** S. Correia Carreira, J. Spencer, W. Schwarzacher, A. M. Seddon

**Affiliations:** a Bristol Centre for Functional Nanomaterials, University of Bristol, Bristol, United Kingdom; b School of Cellular and Molecular Medicine, University of Bristol, Bristol, United Kingdom; c H. H. Wills Physics Laboratory, University of Bristol, Bristol, United Kingdom; University of Bayreuth

## Abstract

In order to identify pathogens rapidly and reliably, bacterial capture and concentration from large sample volumes into smaller ones are often required. Magnetic labeling and capture of bacteria using a magnetic field hold great promise for achieving this goal, but the current protocols have poor capture efficiency. Here, we present a rapid and highly efficient approach to magnetic labeling and capture of both Gram-negative (Escherichia coli) and Gram-positive (Staphylococcus aureus) bacteria using cationized magnetoferritin (cat-MF). Magnetic labeling was achieved within a 1-min incubation period with cat-MF, and 99.97% of the labeled bacteria were immobilized in commercially available magnetic cell separation (MACS) columns. Longer incubation times led to more efficient capture, with S. aureus being immobilized to a greater extent than E. coli. Finally, low numbers of magnetically labeled E. coli bacteria (<100 CFU per ml) were immobilized with 100% efficiency and concentrated 7-fold within 15 min. Therefore, our study provides a novel protocol for rapid and highly efficient magnetic labeling, capture, and concentration of both Gram-positive and Gram-negative bacteria.

**IMPORTANCE** Antimicrobial resistance (AMR) is a significant global challenge. Rapid identification of pathogens will retard the spread of AMR by enabling targeted treatment with suitable agents and by reducing inappropriate antimicrobial use. Rapid detection methods based on microfluidic devices require that bacteria are concentrated from large volumes into much smaller ones. Concentration of bacteria is also important to detect low numbers of pathogens with confidence. Here, we demonstrate that magnetic separation columns capture small amounts of bacteria with 100% efficiency. Rapid magnetization was achieved by exposing bacteria to cationic magnetic nanoparticles, and magnetized bacteria were concentrated 7-fold inside the column. Thus, bacterial capture and concentration were achieved within 15 min. This approach could be extended to encompass the capture and concentration of specific pathogens, for example, by functionalizing magnetic nanoparticles with antibodies or small molecule probes.

## INTRODUCTION

Infectious diseases are among the world's most pressing health challenges, and developing strategies for rapid identification of pathogens is particularly important for effective treatment and for combating antibiotic resistance. However, many current methodologies rely on culture-based microbiological methods that take several days. Rapid identification of pathogens can be achieved by detecting specific genes (1) or by antibody-directed binding of pathogens to a sensor surface (2). Microfluidic devices constitute excellent platforms for cheap and high-throughput implementation of these methods (2, 3). Similarly, hand-held biosensors can rapidly detect and identify bacteria at concentrations as low as 100 CFU per ml (4). However, all of these devices typically handle volumes of 50 to 250 μl, while clinical blood or urine samples usually range between 5 and 20 ml in volume. Therefore, concentration of pathogens from large into small volumes represents an important processing step in microfluidics-based diagnostics. In addition, preconcentration of small amounts of bacteria can aid in their detection and identification (5–7).

**FIG 1:**
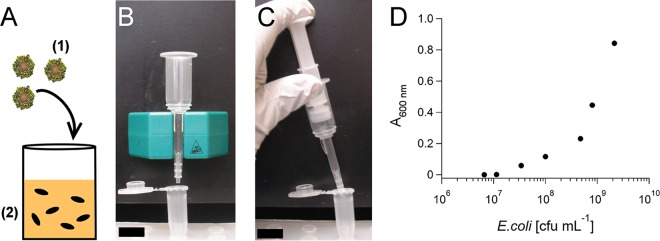
Schematic diagram of the procedure for magnetic labeling and capture of bacteria using cationized magnetoferritin and assessment of optical absorbance as a tool for bacterial quantification. (A) Cationized magnetoferritin (1) is added to a bacterial suspension (2) and incubated for 15 min to achieve magnetization of the cells. (B) The suspension is loaded onto a magnetic separation column, which is attached to a magnet. Magnetized bacteria are immobilized and concentrated in the column. (C) Captured bacteria are eluted by removing the magnet and pushing 0.4 ml of water through the column using a plunger. Bars, 3 cm. (D) Absorbance measured at 600 nm as a function of E. coli present in the water sample as determined by the plate count method. At <10^7^ CFU ml^−1^, absorbance readings are not sensitive enough to accurately determine numbers of cells.

Magnetic labeling of bacteria in suspension enables their extraction from aqueous samples using a magnetic field, such that low numbers of bacteria can be concentrated (8). Previous studies reported the use of magnetic nanoparticles with different surface functionalizations, such as amine and carboxyl groups (9, 10), or small molecules, such as vancomycin (8, 11). In all of these studies, a permanent magnet is placed against a vial containing the magnetized bacteria, which results in sedimentation of the cells. However, capture efficiency varies between 35 and 97%, depending on the surface functionalization (Table 1). Here, we propose a novel approach to capture and concentrate bacteria, which involves the use of cationic magnetic nanoparticles and commercially available magnetic cell separation (MACS) columns; the former enable ultrafast labeling (12), while the latter enhance the efficiency of capture and concentration. Use of MACS columns has traditionally been restricted to the capture of magnetically labeled mammalian cells, and their high capture efficiency has been attributed to the generation of strong magnetic field gradients in the column (13). In this setup, a small plastic column is filled with steel beads and placed against a magnet (Fig. 1B). Cells that have been labeled with superparamagnetic nanoparticles (SPIONs) are immobilized when they are passed through the column in aqueous suspension. Captured cells can be released within a small volume of buffer when the magnet is removed from the column, because the SPIONs demagnetize in the absence of an external magnetic field (Fig. 1C). In this report, we demonstrate that magnetization and concentration of bacteria can be accomplished within 15 min using cationic SPIONs that rapidly attach to anionic domains on the cell surface (12) and MACS columns to subsequently capture and concentrate the magnetized bacteria with up to 100% efficiency.

**TABLE 1:** Comparison of the E. coli capture efficiency presented in this work with previously reported results

SPION surface	Dose	Time	Capture efficiency (%)	*E. coli* detection	Reference/source
Amine	10 μg ml^−1^^*a*^	1 min	99.97	Plate counting	This work
Amine	1 mg ml^−1^	1 min	97	*A*_600 nm_^*b*^	Huang et al. (9)
Carboxyl	2 mg ml^−1^	12 h	35	*A*_600 nm_	Singh et al. (10)
Mannose	2 mg ml^−1^	45 min	88	Microscopy	El-Boubbou et al. (16)
Vancomycin	0.2 μg ml^−1^	60 min	83	Plate counting	Kell et al. (8)

a Iron content calculated for 0.5 μM cat-MF.

b *A*_600 nm_, absorbance measured at 600 nm.

## MATERIALS AND METHODS

### Bacterial culture.

Escherichia coli T7 Express (BL21 derivative; New England BioLabs, USA) transformed with pET45b(+) plasmids (Novagen, Germany) was kindly provided by James Armstrong (School of Cellular and Molecular Medicine, University of Bristol). Staphylococcus aureus SH1000 was a kind gift from Ramesh Wigneshweraraj and Andrew Edwards, Imperial College London (14). E. coli and S. aureus were grown overnight at 37°C in a shaking incubator (200 rotations per min) in 10 ml of lysogeny broth (LB) (BD, Oxford, United Kingdom) (15) or nutrient broth (Lab M, Heywood, United Kingdom), respectively. LB was supplemented with 1 μl per ml broth of a 50 μg ml^−1^ carbenicillin solution (Apollo Scientific, United Kingdom), and nutrient broth was supplemented with 3.4 μg ml^−1^ chloramphenicol.

### Rapidity and extent of E. coli magnetization with cat-MF.

The preparation and detailed characterization of cationized magnetoferritin (cat-MF) have been previously described (12). Briefly, a cobalt-doped iron oxide core was mineralized inside horse spleen apoferritin (Sigma-Aldrich, United Kingdom). The protein surface was cationized by conjugating *N*,*N*′-dimethyl-1,3-propanediamine to acidic amino acid residues via a 1-ethyl-3-(3-dimethylaminopropyl)carbodiimide-mediated cross-linking reaction.

For magnetization experiments, liquid bacterial culture samples were diluted with sterile distilled water (dH_2_O) to achieve an absorbance of approximately 0.3 at 600 nm; 0.3 ml of this suspension was mixed with an equal volume of sterile dH_2_O or 1 μM cat-MF (sterile filtered) to yield a final cat-MF concentration of 0.5 μM. The mixture was agitated briefly and then incubated at room temperature for 1, 5, 15, and 30 min. After each incubation period, 0.5 ml of the bacterial suspension was loaded onto MS MACS columns (Miltenyi Biotec, United Kingdom), and the flowthrough was collected. To determine CFU per milliliter in the bacterial suspension before MACS, samples of the initial bacterial suspension were diluted 1/10^5^, 1/10^6^, and 1/10^7^, and 0.1-ml samples of these dilutions were plated out in triplicate and incubated at 37°C overnight. To determine the number of CFU in the water after MACS, samples of the flowthrough were diluted 1/10 and 1/100, plated out in triplicate, and incubated at 37°C overnight. After incubation, the numbers of colonies on the agar plates were counted, and CFU per milliliter were determined by taking into account the respective dilution factors. The percentage of immobilization was calculated as follows:

(1) $$\text{immobilization}\;(\%)=100-\left(\frac{c_{\text{after}}}{c_{\text{before}}}\cdot 100\right)$$

where *c*_after_ is the bacterial concentration in the flowthrough after MACS and *c*_before_ is the bacterial concentration in the initial bacterial suspension before MACS.

### E. coli magnetization as a function of cat-MF concentration.

Liquid bacterial culture samples were diluted with sterile dH_2_O to achieve an absorbance of approximately 0.3 at 600 nm. A 0.3-ml sample of this suspension was mixed with an equal volume of sterile water (untreated control) or various concentrations of cat-MF (sterile filtered) to yield a final cat-MF concentration of 0.01 to 1 μM cat-MF. The mixture was agitated briefly and then incubated at room temperature for 15 min. After the incubation period, 0.5 ml of the bacterial suspension was loaded onto the MACS column, the flowthrough was collected, and CFU per milliliter and percent removal were determined as described above.

### E. coli magnetization as a function of bacterial number.

Liquid bacterial culture samples were diluted with sterile dH_2_O to achieve an absorbance of approximately 0.3 at 600 nm. This suspension was further diluted in a series of 1 in 10, down to a dilution of 1/10^6^, resulting in a total of 7 samples containing different bacterial numbers. A 0.3-ml sample of each dilution was incubated with an equal volume of 1 μM cat-MF (sterile filtered) to yield a final cat-MF concentration of 0.5 μM. The mixture was agitated briefly and then incubated at room temperature for 15 min. A 0.5-ml sample of the bacterial suspension was loaded onto the MACS column, the flowthrough was collected, and CFU per milliliter in the samples loaded onto the column and in the flowthrough were determined as described above. The number of E. coli immobilized in the column was determined by subtracting the number of E. coli detected in the flowthrough from the number of E. coli loaded onto the MACS column.

### Concentration of small amounts of E. coli.

Liquid bacterial culture samples were diluted with sterile dH_2_O to achieve an absorbance of approximately 0.3 at 600 nm. This suspension was further diluted in a series of 1 in 10, down to a dilution of 1/10^7^. A 1-ml sample of this dilution was incubated with an equal volume of 1 μM cat-MF (sterile filtered) to yield a final cat-MF concentration of 0.5 μM. The mixture was agitated briefly and then incubated at room temperature for 15 min. A 2-ml sample of the bacterial suspension was loaded onto the MACS column, and the flowthrough was collected. The column was removed from the magnet, and any bacteria immobilized in the column were eluted with 0.4 ml of sterile dH_2_O using a plunger. The initial solution, the flowthrough, and the eluted bacterial suspension were plated out without further dilution. CFU per milliliter and percent removal were determined as described in the previous section.

### Comparing magnetic captures of E. coli and S. aureus.

Liquid bacterial culture samples were diluted with sterile dH_2_O to achieve an absorbance of approximately 0.3 at 600 nm, and 0.3 ml of each suspension was mixed with an equal volume of sterile dH_2_O or 1 μM cat-MF (sterile filtered) to yield a final cat-MF concentration of 0.5 μM. The mixtures were agitated briefly and then incubated at room temperature for 15 min. A 0.5-ml sample of each bacterial suspension was loaded onto the MACS column, the flowthrough was collected, and CFU per milliliter and percent removal were determined as described above.

## RESULTS

### Developing a protocol for rapid magnetic labeling of E. coli and highly efficient capture in magnetic separation columns.

**FIG 2:**
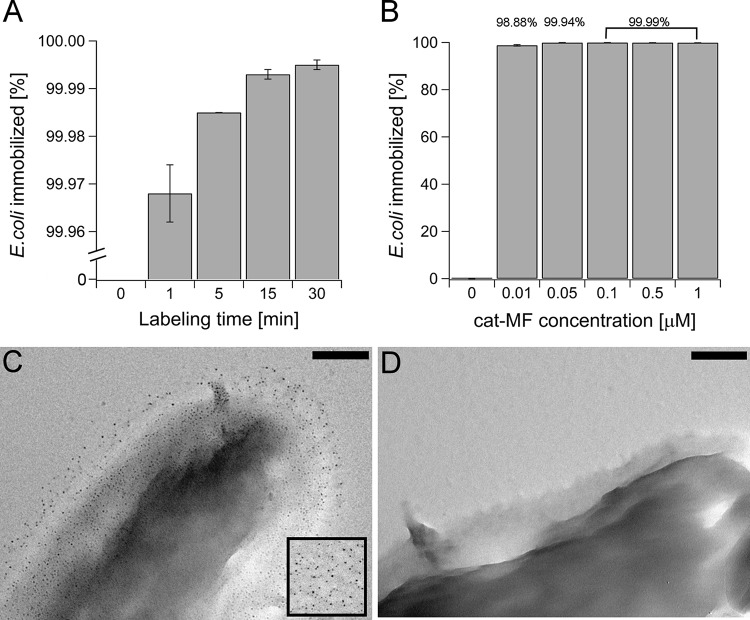
Magnetization of E. coli with cationized magnetoferritin (cat-MF). (A) Percent immobilization of E. coli in magnetic separation columns from water samples inoculated with 10^8^ CFU ml^−1^ after different incubation periods with 0.5 μM cat-MF. (B) Different incubation concentrations of cat-MF for 15 min. Averages and standard deviations from three plate counts are shown. (C) TEM image of E. coli exposed to 0.5 μM cat-MF for 1 min, immobilized, and then eluted from the MACS column. Electron-dense nanoparticles with a diameter matching cat-MF uniformly cover the surface of the bacterium. The inset shows cat-MF particles on their own. (D) Untreated E. coli. No nanoparticles are visible on the surface. Bars, 200 nm.

Rapid isolation of bacteria from aqueous samples represents a first step in the detection of pathogens. Here, we show the potential of cationized magnetoferritin (cat-MF) to rapidly magnetize E. coli. First, magnetic capture efficiency was investigated by incubating 0.6-ml water samples inoculated with approximately 10^8^ CFU ml^−1^ E. coli for 1 to 30 min with 0.5 μM cat-MF and passing the water samples through a MACS column. We found that 99.968% ± 0.006% of E. coli were removed from the water sample by magnetic capture after a 1-min incubation with cat-MF, which increased to 99.995% ± 0.001% after a 30-min incubation period (Fig. 2A). Next, the concentration-dependent magnetization efficiency of cat-MF was investigated by incubation of water samples containing approximately 10^8^ CFU ml^−1^ E. coli with cat-MF concentrations ranging from 0 to 1 μM for 15 min. Untreated E. coli cells were not retained in the MACS column, indicating that cat-MF exposure was necessary to achieve magnetic capture of bacteria. Remarkably, exposure to the lowest cat-MF concentration resulted in the immobilization of 98.8% of E. coli in the MACS column (Fig. 2B). While higher cat-MF concentrations led to the capture of more bacteria, the maximum immobilization efficiency was achieved using 0.1 μM cat-MF, and higher cat-MF concentrations did not improve capture efficiency.

Transmission electron microscopic (TEM) imaging of E. coli eluted from the magnetic column after a 1-min incubation with cat-MF revealed the presence of electron-dense nanoparticles of approximately 5 to 6 nm diameter on the surface of the bacterium (Fig. 2C). It was concluded that these particles were cat-MF, because no nanoparticles were visible in untreated E. coli samples (Fig. 2D) and the size of the particles observed on E. coli corresponded to the size of cat-MF (Fig. 2C, inset).

**FIG 3:**
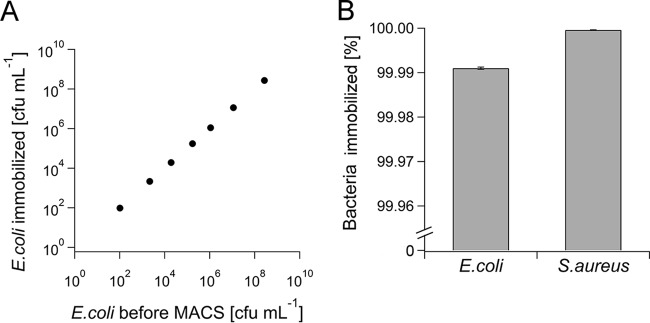
Immobilization efficiency as a function of E. coli concentration and magnetic concentration of low numbers of E. coli. (A) Water samples were inoculated with 1 × 10^2^ to 2 × 10^8^ CFU ml^−1^ E. coli and incubated with 0.5 μM cat-MF for 15 min. The number of E. coli immobilized in the MACS column was linearly proportional to the number of bacteria loaded onto the column. Averages and standard deviations from three plate counts are shown (error bars are too small to be visible). (B) Comparison of magnetic capture efficiencies for E. coli and S. aureus labeled with 0.5 μM cat-MF for 15 min. Averages and standard deviations from three plate counts are shown.

Finally, immobilization efficiency was investigated as a function of bacterial number. Water samples were inoculated with approximately 10^2^ to 10^8^ CFU ml^−1^ E. coli, incubated with 0.5 μM cat-MF for 15 min, and passed through a MACS column. It was found that the number of immobilized E. coli was linearly proportional to the amount of bacteria present in the inoculated sample (Fig. 3A). Immobilization efficiencies were >99.9% in all cases except for the sample containing 10^2^ CFU ml^−1^, for which 100% capture efficiency was recorded.

### Gram-positive bacteria can also be captured rapidly and efficiently.

In a direct comparison of two bacterial species, water samples inoculated with approximately 10^8^ CFU ml^−1^ of E. coli or S. aureus were incubated with 0.5 μM cat-MF for 15 min and passed through MACS columns. It was found that capture efficiency was higher for S. aureus than for E. coli, with 99.99% of E. coli captured in MACS columns compared to 99.9999% of S. aureus (Fig. 3B).

### E. coli is captured and concentrated 7-fold within 15 min.

**FIG 4:**
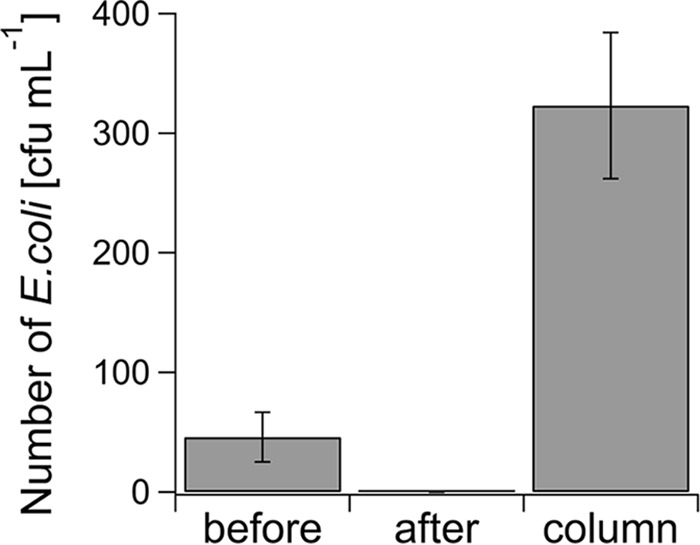
Capture and concentration of dilute E. coli. A 2-ml water sample containing 46 CFU ml^−1^ E. coli (labeled “before”) was incubated with 0.5 μM cat-MF for 15 min. After MACS, no bacteria were detected in the flowthrough (labeled “after”). E. coli cells immobilized in the MACS column were eluted with 0.4 ml sterile water, through which a 7-fold concentration increase of bacteria was achieved (labeled “column”). Averages and standard deviations of three plate counts are shown. Note that although an elution volume of 0.4 ml was applied to the column reservoir, we observed that only approximately 0.33 ml eluted from the column.

We investigated whether low numbers of bacteria, i.e., <10^2^ CFU ml^−1^, could be completely removed from a water sample and concentrated using immobilization in the MACS column, followed by elution into a small volume. A 2-ml water sample containing approximately 50 CFU ml^−1^ E. coli was incubated with 0.5 μM cat-MF for 15 min and passed through a MACS column. No bacteria could be detected in the flowthrough after MACS (Fig. 4). When the column was removed from the magnet and the immobilized bacteria were eluted using 400 μl sterile water, approximately 320 CFU ml^−1^ E. coli were detected, which represents an almost 7-fold increase in the concentration of bacteria.

## DISCUSSION

Given the tremendous potential of magnetic labeling and capture for isolating bacteria, there have been surprisingly few studies that investigated this technique. Compared to previously reported work in this field, our method represents a dramatic improvement in bacterial capture efficiency over conventional methodologies that consist of a permanent magnet placed against a vial (8–11, 16, 17). In such devices, the magnetically labeled bacteria agglomerate in the applied magnetic field, such that the supernatant can be removed and analyzed for residual bacteria. In Table 1, the results from studies using this approach are compared to the results presented here using MACS. Depending on the surface functionalization, the reported capture efficiencies range from 35% for carboxy-functionalized SPIONs (10) to 97% for amine-functionalized SPIONs (9). Here, we were able to immobilize >99.9% of bacteria using cat-MF, which is effectively an amine-functionalized SPION. We believe that the improvement in capture efficiency observed in our experiments is due to the generation of strong local magnetic fields by the steel beads in the MACS column. In contrast, the use of a permanent magnet placed against a vial has the disadvantage that the magnetic field decays rapidly with the distance from the magnet. Therefore, the magnetic field may not be strong enough to agglomerate magnetized bacteria from the entire volume.

The highest capture efficiency reported to date was 97%, which was achieved using amine-functionalized magnetic nanoparticles (9). However, it should be noted that the authors of that study used absorbance at 600 nm to measure capture efficiency, which is a relatively insensitive detection method. We found that absorbance values could only be related to E. coli concentrations at cell densities of >10^7^ CFU ml^−1^ (Fig. 1D), and therefore we chose the plating method as a more sensitive means of calculating the percentage of immobilized bacteria. The plating method was also used in a study by Kell et al., who reported capture efficiencies of 11 to 83%, depending on the surface functionalization of the SPION used for the magnetization of bacteria (8). This shows that our methodology is indeed superior in terms of magnetic capture efficiency.

From the discussion above, it is apparent that surface functionalization of SPIONs is crucial for efficient magnetic labeling and subsequent capture in a magnetic field. Previously, SPIONs have been functionalized with vancomycin to bind to the surface of a variety of bacteria and enable magnetic capture and concentration. Vancomycin is a glycopeptide antibiotic that interacts strongly with the cell surface of Gram-positive bacteria through hydrogen bonding but has also been found to bind to Gram-negative bacteria, presumably due to nonspecific interactions (17). However, vancomycin conjugation onto SPIONs is not trivial, and capture efficiency depends strongly on the orientation of the molecule on the SPION (8). Here, we present a much simpler functionalization protocol, which rendered cat-MF a positively charged SPION. We have previously shown that cat-MF magnetized mammalian cells via electrostatic binding to anionic proteoglycans on the cell surface (12). Thus, we consider it likely that electrostatic interactions also mediate the adsorption of cat-MF onto anionic domains on the bacterial surface. This is consistent with the findings in previous studies, in which cationized ferritin was used as a TEM “stain” to investigate the distribution of anionic domains on the surface of bacteria (18, 19). These domains could be negatively charged polysaccharides in the lipopolysaccharide layer of E. coli, such as hexuronic acid and other acidic sugars (19, 20).

Although the methodology presented here has been shown to be superior to previously reported protocols, it should be noted that 100% capture efficiency was only achieved when small amounts of bacteria were loaded onto the MACS column (Fig. 3A). Therefore, it can be hypothesized that either the MACS column was saturated with bacteria, thus preventing the capture of all of the bacteria introduced, or there was insufficient magnetic material present to adequately magnetize all of the bacteria. The latter hypothesis is unlikely to be true, given that cat-MF concentrations as low as 0.01 μM (50 times less) still resulted in the immobilization of 98.88% of the bacteria (Fig. 2B). Also, we consider that saturation of the MACS column by bacteria is equally unlikely, given the linear relationship between the number of magnetized E. coli loaded onto the column and the number of E. coli immobilized (Fig. 3A). Saturation of the column is expected to generate a flattening of the curve as the number of applied bacteria increases. A final point to consider then is the size and shape of E. coli, which are very different from the size and shape of mammalian cells, for which the MACS column design was originally optimized. Most mammalian cells are round in suspension with a diameter of approximately 10 μm. However, E. coli cells are much smaller and rod shaped, approximately 1 μm long and 0.5 μm wide. The cells of the strain of E. coli used here are also more mobile than mammalian cells because they are flagellated. Therefore, it is possible that a proportion of the cat-MF labeled E. coli cells were able to overcome the magnetic force and escape from the column. A possible argument in favor of this hypothesis is the fact that immobilization of S. aureus, a species that does not possess flagella, was more efficient (Fig. 3B).

The capacity of cat-MF to efficiently label both Gram-positive and Gram-negative bacteria is an interesting result. Bacterial surface compositions vary widely, in particular between Gram-negative and Gram-positive organisms. Gram-positive bacteria, such as S. aureus, lack the outer lipopolysaccharide layer characteristic of Gram-negative species such as E. coli (20). However, S. aureus has been shown to have a negative zeta potential (21); therefore, negative charges are available for electrostatic binding of cat-MF to the cell surface. Furthermore, previous studies have reported that magnetic capture of S. aureus could not be achieved using amine-functionalized (i.e., cationized) SPIONs and instead required small molecule probes to achieve this (11). However, we were able to efficiently magnetize and capture S. aureus using cat-MF (i.e., an amine-functionalized SPION).

We anticipate that methods for magnetic capture of bacteria from aqueous suspension can be applied in the detection and/or removal of organisms from physiological or environmental fluids. While microbial concentrations in these circumstances may vary widely, in many cases capture of pathogens present at low concentrations may be required. We have shown here that MACS columns reached a capture efficiency of 100% for E. coli concentrations of ≤10^2^ CFU ml^−1^. Thus, an example of the application of our methodology is the capture of E. coli, an organism commonly associated with fecal contamination of water supplies (22), from dilute solutions. The isolation of pathogens present at low concentrations may be required. Another potential application of our method is the concentration of bacteria from dilute suspensions for downstream analysis, because it has been shown that detection of bacteria at concentrations of <10^2^ CFU ml^−1^ is notoriously difficult without preenrichment of bacteria through a culture process (23). We have shown here that cat-MF labeling and subsequent capture in MACS columns can indeed concentrate E. coli from very dilute suspensions (Fig. 4). It was previously reported that the lowest concentration of E. coli that could be captured in a magnetic field after incubation with vancomycin-functionalized SPIONs was 10 CFU ml^−1^. This is of the same order of magnitude as the lowest concentration of E. coli captured in our experiments (∼50 CFU ml^−1^), which was achieved using a much more facile surface functionalization.

Our results indicate that our methodology for magnetic labeling may permit the capture of a range of bacteria, due to the nonspecific labeling mechanism. However, it is possible to functionalize magnetoferritin or other SPIONs with antibodies, such that specific pathogens may be labeled (17, 24–26). With the combination of this technique and the MACS column setup, rapid, highly efficient, and selective capture and concentration of individual pathogens might be possible in the future.

In conclusion, we demonstrate that a rapid magnetic labeling technique combined with immobilization of bacteria in MACS columns enables highly efficient capture (up to 100%) and a 7-fold concentration of low numbers of E. coli within 15 min. The use of MACS columns to capture magnetically labeled bacteria yields higher capture efficiencies than conventional magnet-based setups. Furthermore, our approach enables magnetic capture of the Gram-positive bacterium S. aureus with 99.999% efficiency. Thus, magnetic labeling with immobilization in MACS columns is a viable approach for complete capture and rapid concentration of low numbers of bacteria, representing an important step toward fast detection and identification of bacterial pathogens.
